# Otology

**Published:** 1904-08-20

**Authors:** 


### OTOLOGY.

(*Continued from page* 330.)

**Suppuration of the Labyrinth.**—W. Milligan [4] has written recently on this subject. The affection is a very serious one on account of its liability to cause meningitis, and to damage the acoustic or static segments of the internal ear. Its causes are: (1) Extension of suppuration from the middle ear; (2) extension of suppuration along perivascular or perineural sheaths from the base of the brain; (3) infection from the blood stream; and (4) injuries. Some cases run a slow and almost painless course, and lead to the exfoliation of considerable portions of the labyrinth. Such cases are frequently of a tubercular nature. In 60 cases of the affection Hinsberg found the path of infection from the middle ear was through erosion (fistula) of the horizontal semicircular canal in 27, through the fenestra ovalis in 17, through the fenestra rotunda in two, through both apertures in three, through the promontory in seven, and in eight through one of the other semi-circular canals. The septic process may become localised to the region of the canals or vestibule or cochlea, but usually, through contiguity, these parts are simultaneously affected. In one case during a radical mastoid operation, Milligan removed sequestra containing portions of the cochlea and semicircular canals from a man who had had otorrhœa for 20 years, and severe attacks of vertigo. Improvement set in at once after the operation. When the infection passes into the cranial cavity it usually first attacks structures in the posterior fossa, but it may first pass into the middle fossa by way of the superior semicircular canal. Diffuse purulent meningitis is the usual result of the spread of infection inwards, but occasionally a localised inflammation is set up. Probably 20 to 30 per cent. of cerebellar abscesses are secondary to labyrinthine suppuration. The affection may run an acute or chronic course. In the acute cases the symptoms are deep-seated pain, rapid elevation of temperature, sickness or nausea, pronounced vertigo, horizontal or vertical nystagmus, and severe nerve deafness. More usually the course is almost chronic and the symptoms less marked. The onset of facial paralysis in suppurative otitis frequently indicates labyrinthine implication. Where the suppuration is localised the symptoms may be mainly static or mainly acoustic. In diagnosis the chief difficulty arises from the resemblance between the symptoms of labyrinthine suppuration and of cerebellar abscess. Points in favour of the former would be marked nerve deafness, sensation of dizziness rather than the vertigo associated with staggering gait, absence of occipital pain, head retraction, optic neuritis, and somnolency. Diagnostic difficulties are increased by the fact that cerebellar and labyrinthine suppuration may co exist. The prognosis is, of course, grave, owing to the danger of septic meningitis. The treatment consists in performing the ordinary radical mastoid operation and carefully searching, under good illumination, for any fistulous tract leading to the labyrinth; such an opening, if found, is then enlarged, the path of infection followed up, and all granulation tissue and purulent débris removed therefrom.

The Treatment of Aural Vertigo.—R. Lake[5] records a case in which he removed the semicircular canals for this affection with success. The patient, a woman of 21, had been liable to attacks of aural vertigo with sickness and vomiting, and had had gradually increasing deafness and tinnitus, for five years. Lake, during the six months immediately preceding the operation, tried various remedies, including large doses of quinine with hydrobromic acid, iodide, strychnine, belladonna, mercury, and pilocarpine injections, all without success. A radical mastoid operation was performed and the bony opening enlarged forwards, upwards, and backwards. The malleus and incus were removed, and then, with burrs of various sizes, the semicircular canals were removed. The wound was swabbed with Lister's strong solution. After the operation there was severe shock for an hour, then, for 48 hours, the patient lay coiled up like a person with cerebral irritation. For two days the eyelids remained tightly closed, but through the lids rhythmical movements of the eyeballs could be seen. On the tenth day the patient could walk with assistance for a few steps. She could only turn towards the right or sound side without falling at this time. If she turned to the left she fell to the right side. At the end of four weeks she could do anything without falling. Up to the time of reporting the case, 14 weeks, there had been no return of the vertigo. The tinnitus remained as bad as ever, but the hearing power for the voice was considerably improved. T. Wilson Parry[6] gives a full account of a very severe case of paroxysmal aural vertigo (Ménière's disease), in which relief was obtained by inserting a seton into the nape of the neck and by keeping the man from his work.

Abscess of the Brain.—A. L. Whitehead[7] records the case of a woman of 20 years of age upon whom he successfully operated twice for temporo sphenoidal abscesses, the first time on the left side, the second time, two and-a-half years later, on the right side. The writer remarks that many cases are known in which operations have been performed upon two or more abscesses on the same side of the brain, but that he has been unable to find the record of a case in which the temporal lobes on both sides had been destroyed by abscess secondary to ear-disease. At the first operation the patient was profoundly unconscious, with irregular breathing, a pulse-rate of 56, and some rigidity of the right arm. The history was that she had had left-sided headache for three weeks, and that three days before the operation diplopia and mental dulness had developed, and the latter had deepened to complete unconsciousness on the last day. Three and a-half ounces of stinking pus were evacuated from the left temporo-sphenoidal lobe A drain-tube was left in the abscess-cavity for 26 days. Complete recovery followed. At the second operation, two and a-half years later, the patient was in a drowsy state, with intense pain in the right side of the head and a subnormal temperature. An abscess was found in the brain immediately beneath the dura above the tegmen antri. Three ounces of pus were evacuated. The senses of taste and smell have not been affected by the extensive destruction of the lower temporal lobes on both sides, the hearing power remains good and the memory and general mental power are perfect. W. Billington[8] operated on a boy in a comatose state, with stertorous breathing of Cheyne-Stokes type and evacuated two ounces of pus from the left temporo-sphenoidal lobe of the brain. No anæsthetic was used. Full consciousness was regained on the second morning after the operation. The drainage tube was removed on the fourth day. Perfect recovery followed, and the case shows how a seemingly hopeless condition may be recovered from after draining a cerebral abscess.

4 Lancet, Feb. 13. 1904, p. 416. 5 Ibid, June 4, 1904, p. 1567.

6 Ibid, March 5, 1904, p. 649. 7 Ibid, Feb. 13, 1904, p. 428.

8 Brit. Med. Jour., April 9, 1904, p. 8361.

